# Flame-retardant polyvinyl alcohol membrane with high transparency based on a reactive phosphorus-containing compound

**DOI:** 10.1098/rsos.170512

**Published:** 2017-08-09

**Authors:** Sha Peng, Ming Zhou, Feiyan Liu, Chang Zhang, Xueqing Liu, Jiyan Liu, Liyong Zou, Jia Chen

**Affiliations:** Key Laboratory of Optoelectronic Chemical Materials and Devices of Ministry of Education, Flexible Display Materials and Technology Co-innovation Center of Hubei Province, Jianghan University, Wuhan 430056, People's Republic of China

**Keywords:** flame-retardant, polyvinyl alcohol, transparency, phosphorus-containing compounds

## Abstract

Flame-retardant polyvinyl alcohol (PVA) membranes with high transparency and flexibility were prepared by mixing an aqueous solution of a phosphorus-containing acrylic acid (AOPA) with PVA. The reaction between AOPA and PVA, the transparency, the crystallinity and the flexibility of the membrane were investigated with Fourier transform infrared spectrometry (FTIR), UV–vis light transmittance, X-ray diffraction and tensile tests, respectively. The limited oxygen index (LOI) and vertical flame (UL 94 VTM), microscale combustion calorimetry, thermogravimetric analysis (TGA) and TGA-FTIR were employed to evaluate the flame retardancy as well as to reveal the corresponding mechanisms. Results showed that PVA containing 30 wt% of AOPA can reach the UL 94 VTM V0 rating with an LOI of 27.3% and retain 95% of the original transparency of pure PVA. Adding AOPA reduces crystallinity of PVA, while the flexibility is increased. AOPA depresses the thermal degradation of PVA and promotes char formation during combustion. The proposed decomposition mechanism indicates that AOPA acts mainly in the condensed phase.

## 1. Introduction

Polyvinyl alcohol (PVA) is a biocompatible and biodegradable polymer. Good film formation, high transparency and mechanical properties make PVA films widely used in the textile industry, furnishings, adhesives and packaging materials [1–3]. Like most polymers, PVA is flammable, which is a potential hazard to the above-mentioned application. The common solution to improving the flame retardancy of PVA is incorporating flame retardants into the polymer matrix by physical mixing or by chemical reaction [4–8]. No matter what method is adopted, maintaining the transparency and endowing satisfactory flame retardancy of the final material as well put forward more requirements for the flame retardant additives. It is known that transparency of a multicomponent system depends greatly on the compatibility between components or the dispersion of additives in the matrix [9–13]. For the inorganic-based flame-retardant PVA, dispersing additive in the matrix on the nanoscale can maintain the transparency of PVA. However, nanoparticles easily aggregate with each other; it is difficult to disperse them in the polymer at the concentration which meets the flame retardancy requirement. Hence this approach is not available on a commercial scale so far [14–16]. Organic compounds are easier to disperse into polymer than inorganic additives. With regard to the inert organic compounds, they tend to migrate from the polymer during use and ageing. Consequently, performance of the materials with respect to their mechanical and electrical properties and flame retardancy will change gradually with the migration of organic additives [17,18]. Organic additives having an interaction with PVA by chemical bonding or hydrogen bond are a better option to maintain the transparency of polymer when improving the flame retardancy [7,19,20] than the inert organic additives. These reactive additives can mix with the matrix at a molecular level by chemical bonding if the polarity and solution parameters of the additives are close to that of the polymer. Compared with reactive halogen-containing additives, phosphorus-containing compounds are more reliable and safer due to release of non-toxic gases and less smoke during combustion [21–23].

There are lots of phosporus-based flame-retardant PVAs. However, very few publications relate to the reactive phosphorus compound-modified PVA flame-retardant systems which exhibit both excellent transparency and flame retardancy. Wang *et al.* [4] reported a flame-retardant PVA membrane obtained through coating P–Si compounds on the surface of a PVA film. The flame retardant is fixed onto the surface of the film by reacting with the hydroxyl group of the PVA. The final film retains 90% of the transparency of the original PVA and the membrane also passes the UL 94 VTM V0 rating.

In this work, a novel phosphorus-containing acrylic acid (AOPA) was introduced into PVA by mixing PVA/AOPA in water. A chemical connection is formed between AOPA and PVA through the reaction of phosphinic acid of AOPA and the hydroxyl group of the PVA. Flame-retardant performance, flexibility and transparency of the PVA/AOPA membrane were investigated in detail.

## 2. Material and methods

### 2.1. Materials

**Figure 1.**
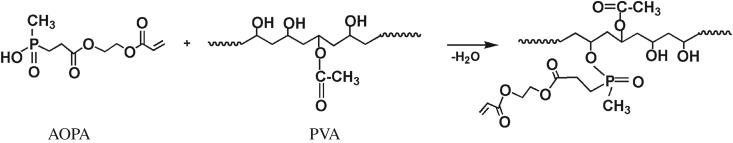
Reaction between PVA and AOPA.

Poly(vinylalcohol), Mowiol® PVA-117 with Mw = 145 000 and an alcoholysis degree of 98% was provided by Aladdin reagent company. AOPA was synthesized in our lab. Its structure and reaction with PVA is shown in figure 1.

### 2.2. Preparation of polyvinyl alcohol/phosphorus-containing acrylic acid membrane

PVA aqueous solution (10 wt%) was obtained by dissolving PVA into water at 90°C for 3 h; then the AOPA of different mass was added with mechanical stirring for about 30 min. A transparent solution of PVA/AOPA was poured onto a glass plate and dried at ambient temperature for 24 h followed by 80°C in vacuum for 8 h. The obtained film is about 0.12 mm. The formulation and samples' names are listed in table 1.

**Table 1.** Formulation of PVA/AOPA membranes and mechanical properties.

	component (wt%)					
sample	PVA	AOPA	elongation at breaking (%)	modulus (MPa)	strength (MPa)	transmittance at 600 nm (%)
PVA	100	0	40.3	964	88.4	90.5
PVA/AOPA20	80	20	102	485	62.7	88.5
PVA/AOPA30	70	30	180	365	52.4	86.9
PVA/AOPA40	60	40	107	476	55.8	86.4

### 2.3. Measurements and characterization

The ^1^H-NMR spectra were recorded in a solution of DMSO-d_6_ at 25°C with a Mercury VX-300 instrument operating at 400 MHz (Varian, US), using TMS as the inner reference. The Fourier transform infrared (FTIR) spectra were recorded on a Nicolet iS10 FTIR spectrometer; samples of about 10 mg were heated in aluminium pans from 40°C to 700°C at a heating rate of 1°C s^−1^. The flow rate of N_2_ and O_2_ was 80 ml min^−1^ and 20 ml min^−1^, respectively. Tensile property testing was carried out using a CMT6000 universal testing machine (SANS, China) following the standard ASTM D638. The testing speed was 10 mm min^−1^. Thermogravimetric analysis (TGA) was carried out with the TSDT Q600 thermogravimetric analyser (TA, USA). Samples of about 10 mg were heated in aluminium pans from room temperature up to 700°C at a heating rate of 20°C min^−1^ in N_2_. TGA was coupled with a Nicolet iS10 FTIR spectrometer (Thermo Fisher Scientific, USA). All the gas flow produced from TGA was transferred to the infrared analysis cell through a transfer tube having an inner diameter of 1 mm. The transfer line and the gas cell were heated to 200°C. Spectra were obtained *in situ* during the thermal degradation of the sample.

## 3. Results and discussion

### 3.1. The characterization of polyvinyl alcohol/phosphorus-containing acrylic acid

**Figure 2:**
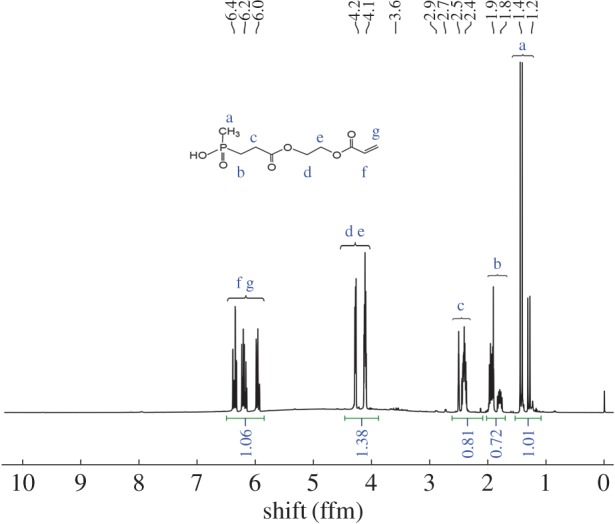
^1^H-NMR spectrum of AOPA.

The structure and ^1^H-NMR spectrum of AOPA is shown in figure 2. The shifts corresponding to all protons of AOPA are: 1.2–1.4 ppm (P–CH_3_); 1.76–2.43 ppm (P–CH_2_–); 2.2–2.5 ppm (C–CH_2_–C=O); 4.1–4.2 ppm (O–CH_2_–CH_2_–O); 5.93–6.39 ppm (–CH=CH_2_). AOPA dissolves in water and shows a good compatibility with PVA.

**Figure 3.**
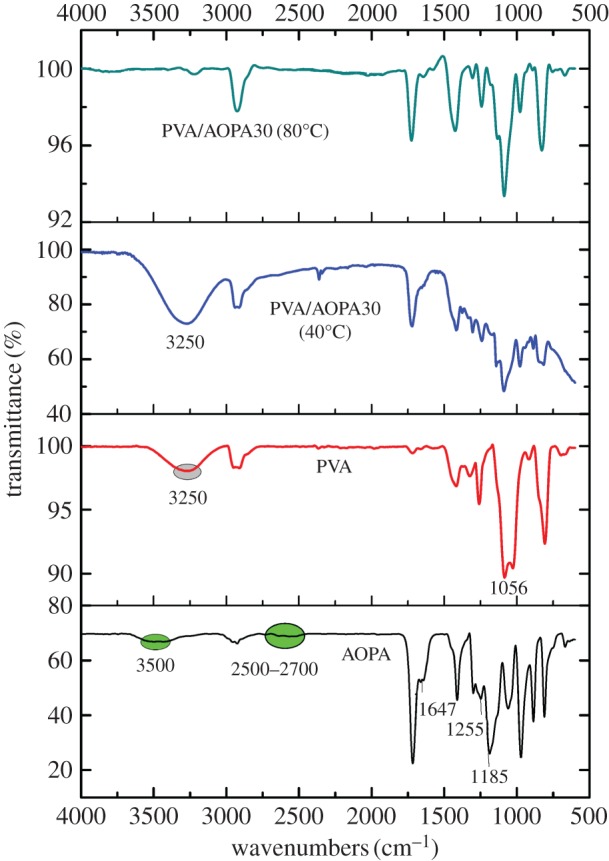
FTIR spectra of PVA, AOPA and PVA/AOPA30 membranes.

FTIR spectra of AOPA and PVA/AOPA30 membranes are shown in figure 3. The characteristic absorption for the AOPA are: 3500 cm^−1^, 2700–2500 cm^−1^ and 977 cm^−1^ (phosphorus acid, O=P–OH), 1725 cm^−1^ (C=O), 1635 cm^−1^ (C=C), 2940, 1400, 890 and 800 cm^−1^ (CH_3_ and CH_2_), 1255 cm^−1^ (P–CH_3_), 1185 cm^−1^ (P=O), 1050 cm^−1^ (C–O–) [24]. The characteristic absorption for the PVA is 3250 cm^−1^ (–OH), 1050 cm^−1^ (C–O). In the spectra of PVA/AOPA30 dried at 40°C, the absorption for the –OH is very strong. After the film is treated at 80°C for 8 h, the absorption at 3250 cm^−1^ becomes weak. The characteristic absorptions at 2500–2700 cm^−1^ and 3500 cm^−1^ for AOPA have vanished. FTIR results have proved the reaction between phosphinic acid of AOPA and the hydroxyl group of PVA. In addition, it is noted that the absorption at 1635 cm^−1^ for the C=C of AOPA becomes weaker after the PVA/AOPA membrane is treated at 80°C, indicating an addition reaction of the AOPA itself has occurred within the membrane.

### 3.2. UV–visible analysis of membranes

**Figure 4.**
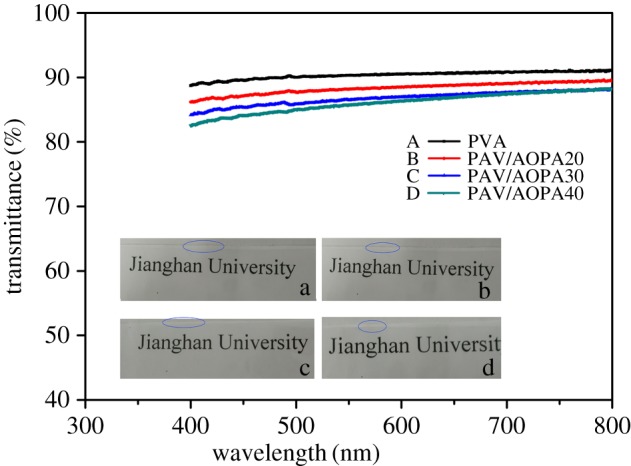
UV–vis transmittance spectra and photographs of membranes.

UV–visible transmittance and photographs of the membranes covering the words are presented in figure 4. In the photographs, the boundary between background and the membrane is marked with a blue ellipse. The words ‘Jianghan University’ under the membranes are clearly visible. The transmittance of the membranes at 600 nm is listed in table 1. In the UV–vis spectra, each membrane possesses a high transmittance of above 85% over a range of 400–800 nm. The transmittance of the membranes drops only by 4.5% when the AOPA content increases from 0 to 40% at 600 nm. High retention of transmittance implies good compatibility between AOPA and PVA.

### 3.3. Tensile properties of membranes

**Figure 5.**
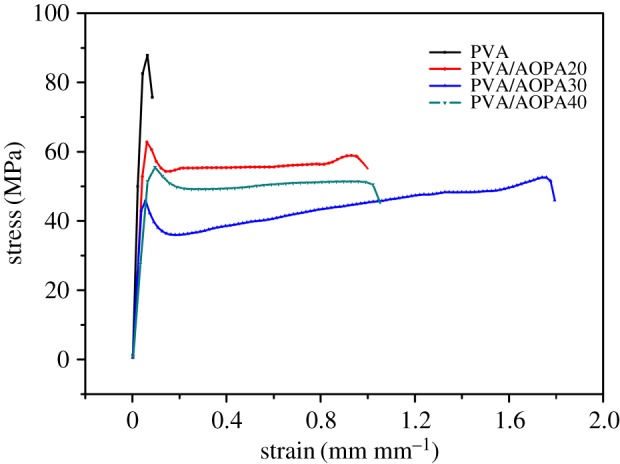
Tensile stress–strain curves for PVA/AOPA membranes.

Figure 5 exhibits the typical tensile stress–strain curves of PVA/AOPA membranes. The average value of tensile strength, tensile modulus and elongation at breaking for five samples are presented in table 1. Adding AOPA makes the PVA membrane more flexible. The elongation at breaking of the membranes increases, while the modulus and the strength decrease with the increase in AOPA content. In the PVA/AOPA, AOPA molecules graft onto PVA as a side chain, the bulk side group enlarges the distance of PVA chains and movement of the polymer chains becomes easy. So low modulus is a result.

### 3.4. X-ray diffraction analysis of polyvinyl alcohol and polyvinyl alcohol/phosphorus-containing acrylic acid membranes

**Figure 6.**
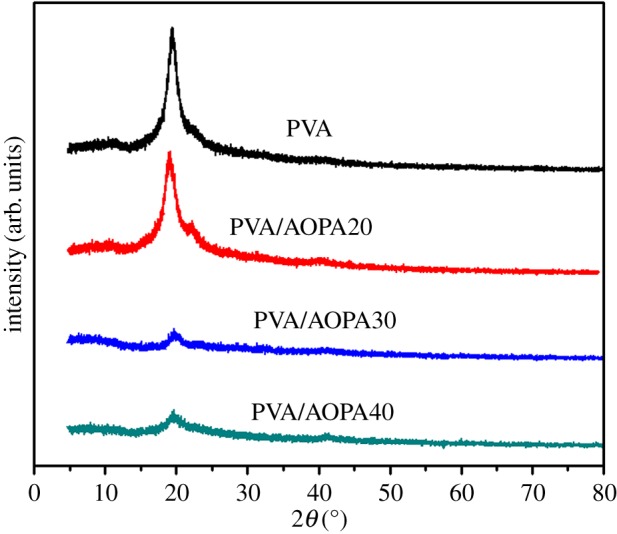
XRD profiles of PVA and PVA/AOPA membranes.

Figure 6 shows the XRD profiles of PVA and PVA/AOPA membranes. PVA exhibits the crystalline structure with diffraction peaks at 19.2°. Incorporating AOPA reduces the crystallinity of PVA, and the diffraction intensity of PVA/AOPA becomes smaller with the increase in AOPA content. After the AOPA content has increased beyond 30%, the diffraction intensity does not decrease any more. The XRD results support the mechanical properties of membranes: Reduction in the crystallinity of PVA is another reason for the higher flexibility of PVA/AOPA membranes. In addition, PVA/AOPA40 shows the same weak intensity as PVA/AOPA30 in the XRD, but the modulus of the membrane is higher than PVA/AOPA30. The reason is the probability that the chains' movement becomes difficult again due to too much AOPA existing as side chains in the PVA/AOPA40 membrane.

### 3.5. Combustion characteristics: limited oxygen index, vertical flame and microscale combustion calorimetry

PVA easily burns in air with flammable dripping. The limited oxygen index (LOI) values and the UL94 test are summarized in table 2. The LOI value of PVA is 19.6%. Adding 20 wt% of AOPA makes a membrane passing the UL 94 VTM V1 rating with LOI 24.8%. When AOPA is 30 wt%, the membrane passes the UL 94 VTM V0 rating with the LOI 28.7%, and combustion time of both *t*_1_ and *t*_2_ is less than 10 s.

**Table 2.** MCC, UL 94 test and LOI data for PVA/AOPA membranes.

sample	*t*_1_/*t*_2_ (s)	UL 94 VTM	LOI (%)	PHRR (W g^−1^)	THR (kJ g^−1^)	*T*_p_ (°C)	HRC (J g^−1^ K^−1^)
PVA	—	/	19.6	344	23.5	307	275
PVA/AOPA20	14.6/12.5	V-1	24.8	221	22.2	444	186
PVA/AOPA30	5.8/4.3	V-0	27.3	209	20.7	447	166
PVA/AOPA40	2.2/1.2	V-0	29.9	187	20.2	438	153

**Figure 7.**
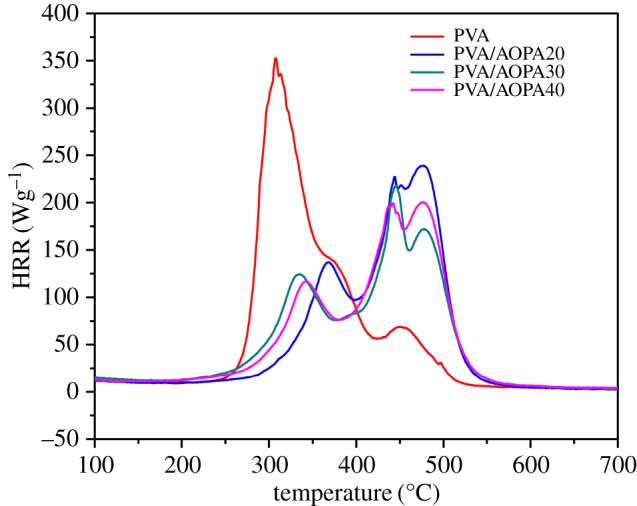
MCC curves for PVA and PVA/AOPA membranes.

Figure 7 presents the heat-release rate (HRR) of PVA/AOPA membranes with temperature by microscale combustion calorimetry (MCC). Data from the MCC test including the peak of heat-release rate (PHRR), the temperature of PHRR (*T*_p_) at the main heat-release stage, the total heat release (THR) and heat-release capacity (HRC) are listed in table 2. All samples show two-step heat release. For the pure PVA membrane, a big heat-release peak appears at 307°C, followed by a minor one around 450°C. For the PVA/AOPA membranes, the heat release at low temperature is depressed and the majority of heat is released at high temperature with *T*_p_ around 430–450°C. In addition, the THR and HRC values of PVA/AOPA membranes are lower than that of PVA and decrease with the increase in AOPA content. HRC is the ratio of specific HRR to the rate of the temperature rise of a sample polymer during a test. It is an important parameter for the determination of fire safety and flame retardancy. The above results imply that adding AOPA moves the heat release of the PVA to a higher temperature. The heat-release speed and the THR of PVA are also reduced in the presence of AOPA.

### 3.6. Thermal stability of polyvinyl alcohol/phosphorus-containing acrylic acid membranes

**Figure 8.**
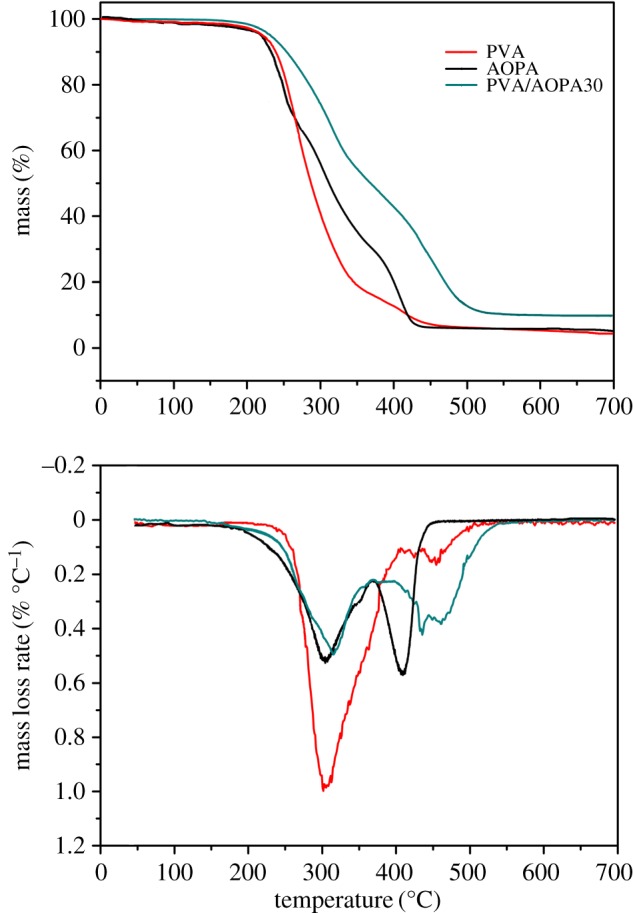
TG-DTG curves of AOPA, PVA and PVA/AOPA in N_2_.

Figure 8 shows the thermogravimetry and differential thermogravimetry (TG-DTG) curves of AOPA, PVA and PVA/AOPA30 in N_2_. Detailed data including temperature at 5% mass loss (*T*_5%_), temperature for the maximum decomposition rate (*T*_max_), the maximum decomposition rate (DTG_max_) and char yields at 700°C are summarized in table 3. All the samples show two-step decomposition. The *T*_5%_ and *T*_max_ of PVA and AOPA in the first stage are well matched to each other. For example, the *T*_5%_ and *T*_max_ in the first stage for the PVA are 240°C and 292°C, respectively; the *T*_5%_ and *T*_max_ in the first stage for AOPA are 237°C and 303°C, respectively. Synchronous decomposition of AOPA and PVA makes the AOPA play a maximum role in trapping the radicals releasing from the PVA or in acting in the condensed phase to promote char formation. Comparing with pure PVA, the thermal degradation of PVA/AOPA30 is greatly depressed in the first decomposition stage (less than 370°C). The char yield obtained at 700°C (9.90%) is twice as much as the calculated value (4.9%). TG-DTG analyses indicate that the presence of AOPA depresses the decomposition of PVA.

**Table 3.** TGA and DTG data for AOPA, PVA and PVA/AOPA30 membrane in N_2._

		I stage (240–370°C)			II stage (370–550°C)			
sample	*T*_5%_ (°C)	mass loss (wt%)	DTG_max_ (% °C^−1^)	*T*_max_ (°C)	mass loss (wt%)	DTG_max_ (% °C^−1^)	*T*_max_ (°C)	char at 700°C (%)
AOPA	237	52.3	0.51	303	41.7	0.57	428	5.95
PVA	240	85.0	1.74	292	10.50	0.16	455	4.50
PVA/AOPA30	242	56.2	0.53	321	34.8	0.43	435	9.90 (4.95)

### 3.7. Analysis of decomposition products

**Figure 9.**
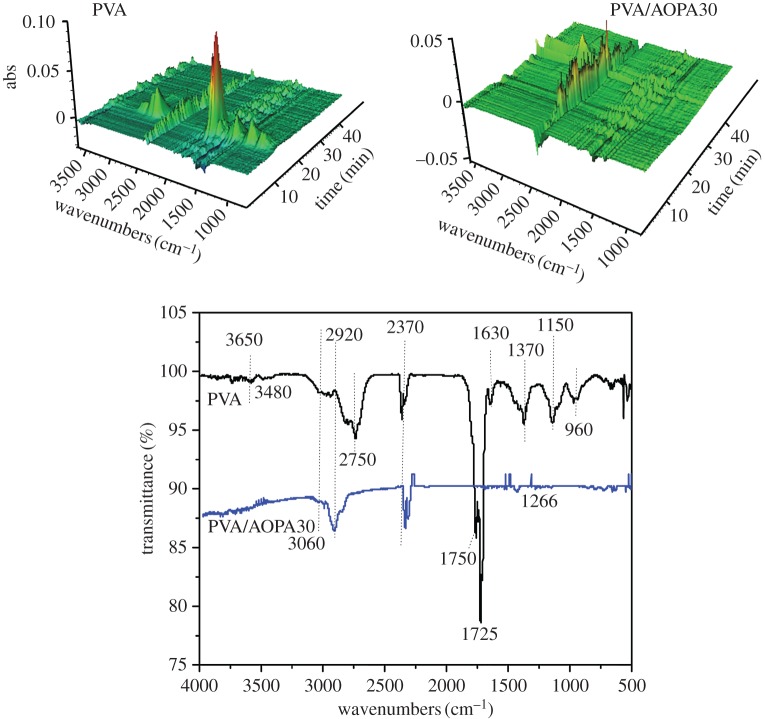
FTIR spectra for pyrolysis gas of AOPA and PVA/AOPA30 in N_2_.

To understand the decomposition behaviour of the membranes, real-time FTIR was used to analyse the volatility during thermal decomposition. Figure 9 shows the 3D-FTIR spectra of the pyrolysis gas, the intensity of the total gas release with time and the 2D-FTIR spectra of pyrolysis gas at the maximum decomposition rate. The gas release of PVA/AOPA30 is much less than that of the pure PVA membrane. The main signals for the gas from PVA are 3650 cm^−1^ (water), 3480 cm^−1^ (–OH), 2920 cm^−1^ (–CH_2_, CH_3_), 2358 and 2170 cm^−1^ (CO_2_), 1750 and 1725 cm^−1^ (C=O), 2750 cm^−1^ combining 1725 cm^−1^ (carboxylic acid), 1622 cm^−1^ (C=C) and 1129 cm^−1^ (C–O). In the PVA/AOPA30 membrane, the signals for the gas containing C=O and C=C group were not observed and the absorptions for –CH_2_, CH_3_ and CO_2_ are weaker than that for the pure PVA membrane.

**Figure 10.**
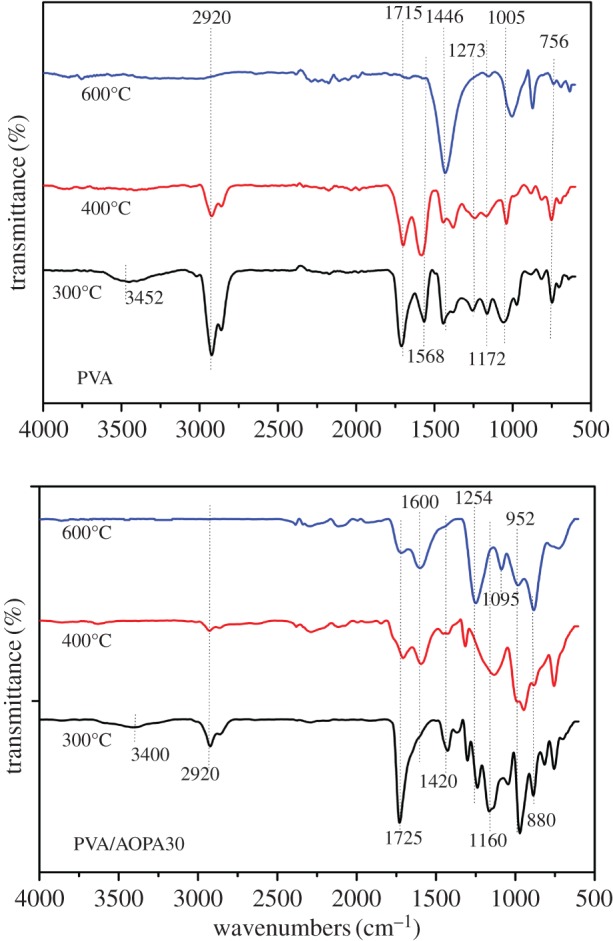
FTIR spectra for the residues of PVA and PVA/AOPA collected at 300°C, 400°C and 600°C in the TG test.

Figure 10 shows the spectra of residues of the PVA and PVA/AOPA30 membrane collected at 300, 400 and 600°C in the TG test. In the spectrum of PVA, peaks at 3452 cm^−1^ (OH), 2920 cm^−1^ (CH_2_, CH_3_), 1172 and 1715 cm^−1^ (C=O) and 1568 cm^−1^ (C=C) decreased when the temperature increases to 400°C, and then these absorptions vanish at 600°C. There is a strong and broad absorption at 1446 cm^−1^ appearing at 600°C which belongs to the conjugated C=C bonds. In the spectrum of PVA/AOPA30 residues obtained at 600°C, a broad and strong absorption at 1254 cm^−1^ is ascribed to the overlapping of signals of C–O–C (about 1100 cm^−1^) with P=O (1300 cm^−1^). The absorption at 1095 cm^−1^ is ascribed to the P–O group. The absorptions at 1600 cm^−1^ for C=C and 1725 cm^−1^ for C=O are retained.

### 3.8. Mechanisms of flame retardancy

**Figure 11.**
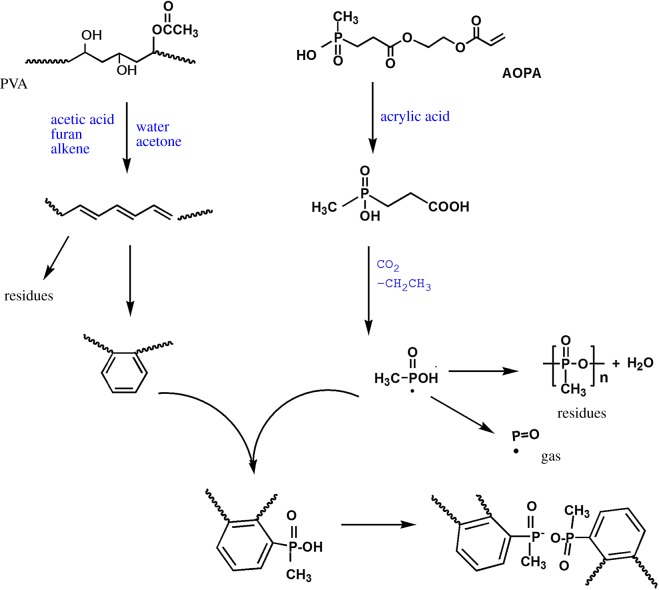
Possible decomposition mechanisms for the PVA/AOPA membrane.

Based on the results of thermal analysis, evolved gas and residues of AOPA, PVA and PVA/AOPA30 and the literature [25–28], a decomposition mechanism as shown in figure 11 is proposed. AOPA undergoes ester-linkage breaking at first producing 2-carboxyethylmethylphosphinic acid during the decomposition of the membrane. 2-carboxyethylmethylphosphinic acid undergoes progressive scission of the P–C bond giving the methylphosphinic acid radical, which can form stable phosphorus acid anhydride R–P(=O)–O–(O=)P–R, or react with aromatic compounds forming the stable phosphate in the residues. PVA also undergoes ester-linkage breaking at first, and progressive degradation releases CO_2_, alkene, hydrocarbon, acid, ester and ether into the gas phase. In the solid phase, unsaturated vinyl compounds are left. Vinyl compounds further form the aromatic compounds and stay in the residues. In the PVA/AOPA, the reaction of methylphosphinic acid radicals from 2-carboxyethylmethylphosphinic acid with aromatic compounds unavoidably occurs and produces extra residues to slow down the heat transfer and depress the progressive degradation of the inner material. So, higher char yield and lower heat release are the results.

## 4. Conclusion

Flame-retardant PVA membranes with high transparency and flexibility were prepared successfully by blending PVA with AOPA. AOPA is immobilized onto the side chain of the PVA through chemical bonds between P–OH and –OH of PVA. The existence of P–O–C was confirmed by FTIR. Adding AOPA reduces the crystallization and enlarges the chain distance of PVA. So, PVA/AOPA membranes show good flexibility. PVA/AOPA membranes have better anti-dripping properties and a lower HRR during combustion than pure PVA. Combining the results from thermal analysis, real-time FTIR spectra of evolved gas and residues during decomposition, a decomposition mechanism is proposed to explain the role of AOPA in improving the flame retardancy and depressing the decomposition of the membrane. The segments from AOPA react with compounds from PVA to form an extra stable char layer, which slows down the progressive spreading of heat and fire.

## Supplementary Material

Synthesis process of AOPA

## Data accessibility

All data used in this research are included in figures, tables and the electronic supplementary material.
