# Supplementary material for: Flame-retardant polyvinyl alcohol membrane with high transparency based on a reactive phosphorus-containing compound
Source: R Soc Open Sci. 2017 Aug 9;4(8):170512. doi: 10.1098/rsos.170512 (PMC5579114; doi:10.1098/rsos.170512)
Supplement: Synthesis process of AOPA [file rsos170512supp1.docx]

The details for the synthesis of AOPA is as following:

***

***

Scheme for synthesis of AOPA

1. ***Experimental Materials***

2-methyl-2, 5-dioxo-1, 2-oxaphospholane (OP) with purity above 98% was provided by Zhenghao Chemical Ltd. (Wuhan, China). hydroxyethyl acrylate (HEA). Cation exchange resin catalyst was obtained from Nanjing Wali Chemical Ltd. (Nanjing, China). cyclohexane acetone, 4A molecular sieve were purchased from Sinopharm Chemical Reagent Ltd. (Beijing, China) used as received.

***2. Synthesis of β-carboxylethylmethylphosphinic acid (CEP)***

To a solution of 0.2 mol (26.8 g) OP in 100 mL of acetone, 0.2 mol (3.6 g) water was added dropwise within 30 min at 50 ^o^C. After the addition was completed, the mixture was re-fluxed for 2 h at 60 ^o^C. Then white CEP precipitated from the solution as the temperature cooled down. The solid CEP was filtered off and washed thrice, with 100 mL of acetone each time. Obtained product is dried at 100 ^o^C for 24 h (Yield =93 %).

***2.3. Synthesis of AOPA***

To a three-neck flask fitted with a reflux condenser, a nitrogen inlet and a stir bar, the mixture of 0.11 mol CEP (16.7 g), 0.116 mol (13.4 g) HEA,3.6 g of cation exchange resin as catalyst and 6 g of quinol as inhibitor in 150 ml of cyclohexane were added. The mixture was kept at 100 ^o^C under refluxing for 5 h. The cation exchange resin was removed by filtering. The solvent and unreacted HEA were removed by distillation under 0.13 MPa pressure. The quinol and unreacted CEP were removed by deionized water. A colorless liquid named AOPA was obtained after removing the trace water by 4A molecular sieve desiccants (Yield=96% ).
